# Supplementary material for: The ChatGPT Effect: Investigating Shifting Discourse Patterns, Sentiment, and Benefit–Challenge Framing in AI Mental Health Support
Source: Behav Sci (Basel). 2025 Aug 28;15(9):1172. doi: 10.3390/bs15091172 (PMC12466438; doi:10.3390/bs15091172)

The ChatGPT Effect: Investigating Shifting Discourse Patterns, Sentiment, and Benefit-Challenge Framing in AI Mental Health Support

Supplemental Materials

Sanguk Lee [1,2,*], Minjin (MJ) Rheu [3] and Jie Zhuang [1]

1. Department of Communication Studies, Texas Christian University, Fort Worth, TX 76109, USA

2. Division of Media & Communication, Hankuk University of Foreign Studies, Seoul 02450, Republic of Korea

3. School of Communication, Loyola University Chicago, Chicago, IL 60660, USA

*Correspondance: lswook555@gmail.com

Table S1. List of Reddit Communities.

Communities	Types	Members
r/CharacterAI	Language Models	1600000
r/ArtificialIntelligence	Language Models	759000
r/ChatGPT	Language Models	7100000
r/OpenAI	Language Models	1800000
r/singularity	Language Models	3200000
r/artificial	Language Models	926000
r/ClaudeAI	Language Models	73000
r/replika	Language Models	79000
r/AI_application	Language Models	11000
r/generativeAI	Language Models	97000
r/LocalLLaMA	Language Models	231000
r/MistralAI	Language Models	66000
r/GoogleGeminiAI	Language Models	8000
r/GeminiAI	Language Models	42000
r/mentalhealth	Mental Health	490000
r/MentalHealthSupport	Mental Health	51000
r/derepression	Mental Health	1000000
r/Anxiety	Mental Health	714000
r/mentalhealthisland	Mental Health	8000
r/hopefulmentalhealth	Mental Health	1900
r/Antipsychiatry	Mental Health	49000
r/SuicideWatch	Mental Health	501000
r/depression_help	Mental Health	99000
r/CPTSD	Mental Health	307000
r/MentalHealthBuddies	Mental Health	1700
r/HolisticMentalHealth	Mental Health	5100
r/bipolar	Mental Health	240000
r/radical_mental_health	Mental Health	12000
r/mentalhealthadvice	Mental Health	1700
r/HealthAnxiety	Mental Health	121000

r/mentalillness	Mental Health	153000
r/anxietyhelp	Mental Health	168000

Table S2. Cross-Referencing Keywords for Data Collection.

Community Types	Keywords
AI Community	'mental health' OR mental-health OR anxiety OR anxieties OR depression OR depressions OR therapy OR therapies OR support OR supports OR stress OR PTSD OR 'panic attack' OR 'panic attacks' OR loneliness OR isolation OR burnout OR wellness OR 'emotional well-being' OR 'mental well-being' OR ‘self-help’ OR self-care OR 'coping mechanisms' OR 'emotional support'
Mental Health Community	chatgpt OR 'chat gpt' OR gpt OR gpt-3 OR gpt-4 OR gpt3 OR gpt4 OR bot OR chatbot OR 'chat bot' OR openai OR 'open ai' OR ai OR a.i. OR 'artificial intelligence' OR llama OR llama2 OR llama3 OR llama3.1 OR alpaca OR claude OR claude2 OR claude3 OR claude3.5 OR character.ai OR 'character ai' OR characterai OR bard OR lamda OR replika OR woebot OR wysa OR cleverbot OR mitsuku OR 'ai dungeon' OR bloom OR ernie OR palm OR anthropic OR cohere

Table S3. Coding Instruction Prompt Used for the Fine-Tuned GPT-4o Model.

You are an expert in content analysis. You will be provided with a single Reddit post that may discusses the use of AI in the context of mental health. Your task is to carefully read the post and code it according to the variables below. Do not make inferences beyond what the instructions specify.<br><br>1. **relevance**:<br>- **Definition:**<br>Determine whether the post discusses AI in a context related to mental health or emotional well-being.<br>- **Criteria for “yes”:**<br>- The post focuses on any interaction with AI tools that might affect emotional states, regardless of the tool’s intended purpose.<br>- The post highlights specific mental health impacts of these interactions (e.g., reducing loneliness, causing distress) or explicitly identifies AI tools designed for mental health support.<br>- **Criteria for “no”:**<br>- The post discusses AI without any connection to mental or emotional health.<br>- The post is purely promotional or solely advertises services or applications.<br>- The majority of the content is a prompt or instruction for a chatbot.<br>- **Criteria for “unknown”:**<br>- There is insufficient information to determine whether the discussion of AI relates to mental or emotional well-being (e.g., the post is too brief or the connection is ambiguous).<br>**Possible values:** "yes", "no", "unknown"<br><br>2. **experience:**<br>- **Definition:** Indicates whether the author directly interacts with AI tools that affect their mental or emotional well-being.<br>- **Criteria:**<br>- **"yes":** The author describes using or having used AI-based tools (e.g., chatbots, AI companions) and acknowledges some impact on their own mental or emotional state.<br>- **"no":** The author discusses only the concept or knowledge of AI’s impact on mental health generally, or references others’ experiences, without mentioning their personal interaction.<br>- **"unknown":** It is unclear if the author directly uses or interacts with such AI tools.<br>- **Possible values:** "yes", "no", "unknown"<br><br>3. **dominant_sentiment_toward_ai:**<br>- **Definition:** The author’s dominant sentiment toward AI applications in the context of mental health or emotional well-being.<br>- **Criteria:**<br>- **"positive":** Predominantly favorable or beneficial views.<br>- **"negative":** Predominantly unfavorable or harmful views.<br>- **"mixed":** Both positive and negative views expressed to a similar degree.<br>- **"neutral":** Observations stated without clear positive or negative sentiment.<br>- **Possible values:** "positive", "negative", "mixed", "neutral"

4. **outcome:**
- **Definition:** The reported impact of AI interaction on the author’s or others’ mental health or emotional well-being.
- **Criteria:**
  - **"positive_outcome":** The AI interaction improved their mental/emotional state.
  - **"negative_outcome":** The AI interaction worsened their mental/emotional state.
  - **"both":** The author explicitly states both improvements and worsening in their mental/emotional state.
  - **"unknown":** No specific outcome is mentioned.
- **Possible values:** "positive_outcome", "negative_outcome", "both", "unknown"

5. **promotion_or_prompt:"
- **Definition:** The post primarily promotes AI applications or services, encourages users to test them, or shares AI-generated prompts without adding personal opinions or critical discussion.
- **Criteria:**
  - **"yes":** The post explicitly promotes AI applications or services, asks users to try them, or shares AI-generated prompts without personal evaluation.
  - **"no":** The post does not focus on promotion or prompting AI use.
- **Possible values:** "yes", "no"

6. **ai_application:"
- **Definition:** language-based AI tools and systems (e.g., chatbots, AI companions, AI-based conversational agents) that interact with humans through natural language.
- **Criteria:**
  - **"character-based chatbot":** Code the post as character-based chatbot, if it mentions character-based ai applications such as character.ai, replica, or simliar systems.
  - **"llms":** Code the post as llms, if it mentions large language models such as gpt, llama, claude, gemini, grook, deepseek, and etc.
  - **"therapy chatbots":** Code the post as therapy chatbots, if it mentions ai applications specifically designed for health care.
  - **"others":** Code the post as others, if it mentions other ai applications. Or information is insufficient to identify the type of ai application.
- **Possible values:** "character-based chatbot", "llms", "therapy chatbots", "others"

7. **accessibility_and_availability**: Refers to the ease of access to AI support and its round-the-clock availability. This includes the convenience of using AI anytime and anywhere, making it particularly helpful for individuals who may not have regular access to human support or mental health resources.
- **Possible values:** "yes", "no"

8. **assisting_self_reflection**: Refers to AI’s ability to encourage users to reflect on their own thoughts, emotions, and behaviors. This includes instances where AI prompts users to think more deeply about their experiences or provides structured feedback that aids personal insight and growth.

- **Possible values:** "yes", "no"

9. **companionship**: Refers to AI's ability to provide a sense of presence or companionship, helping individuals feel less lonely. This includes instances where users perceive the AI as a "friend" or someone they can regularly talk to, fostering a sense of connection. Note that companionship emphasizes the quality of emotional engagement and relational depth rather than mere logistical convenience.
- **Possible values:** "yes", "no"

10. **emotional_support**: Refers to AI's capacity to provide comfort, empathy, and encouragement, helping users feel understood and supported. This includes scenarios where AI responses are perceived as caring, validating, or calming, offering emotional reassurance.
- **Possible values:** "yes", "no"

11.**non_judgmental_nature**: Refers to AI's neutrality and lack of judgment, making users feel comfortable expressing themselves openly without fear of being judged. This aspect is especially beneficial for those hesitant to share with others due to social stigma or fear of judgment.
- **Possible values:** "yes", "no"

12.**personalized_advice_and_support**: Refers to AI's capacity to provide tailored responses based on individual needs or preferences, which may include recommendations or strategies that align with a user's personal situation. This also includes instances where AI adjusts responses to match a user's specific emotional state or personal history, making the interaction feel more relevant and personalized.
- **Possible values:** "yes", "no"

13.**positive_others**: Refers to positive aspects of using AI in a mental health context that are not covered by the above categories.
- **Possible values:** "yes", "no"

14.**adverse_effects**: Refers to negative outcomes caused by AI interactions, such as psychological harm, relational distortions, or emotional destabilization. This includes emotional harm (e.g., gaslighting, emotional invalidation, or reinforcing harmful beliefs), relational harm (e.g., fostering parasocial dynamics, normalizing toxic interaction patterns, or creating unrealistic expectations of human connection), and ethical risks (e.g., exploitative design choices that prioritize engagement over user well-being).
- **Possible values:** "yes", "no"

15.**lack_depth_and_emotional_connection**: Refers to AI's failure to engage in emotionally resonant, contextually aware dialogue. This includes superficiality, lack of empathy, and inability to sustain relational reciprocity or adapt to personal/user-specific context.
- **Possible values:** "yes", "no"

16.**lack_human_like_interaction**: Refers to AI's failure to replicate the natural flow, spontaneity, and social richness of human communication. This includes robotic

conversational patterns, absence of nonverbal cues, and rigid adherence to scripts.
- **Possible values:** "yes", "no"

17.**lack_professional_qualifications**: Refers to AI's lack of professional expertise or qualifications, leading to inappropriate or unhelpful responses, particularly in the context of mental health. This also includes ethical concerns regarding AI's role in providing emotional support without appropriate oversight.
- **Possible values:** "yes", "no"

18.**over_reliance**: Refers to behavioral dependence on AI as a substitute for human agency or connection. This includes addictive patterns (e.g., neglecting responsibilities, sleep, or self-care due to excessive AI interaction), social substitution, or cognitive bypass (e.g., overtrusting AI for decision-making or emotional regulation, eroding critical thinking or self-reliance).
- **Possible values:** "yes", "no"

19.**technical_privacy_issues**: Refers to technical issues (e.g., abrupt disconnection, instability of the program) and privacy concerns (e.g., anxiety related to sharing personal information with AI). This also includes trust issues with AI providers, concerns about data safety, and a lack of transparency regarding data handling.
- **Possible values:** "yes", "no"

20.**negative_others**: Refers to negative aspects of using AI in a mental health context that are not covered by the above categories.
- **Possible values:** "yes", "no"

Table S4. Summary of Chi-Square Tests between AI applications and Benefit and Challenge Variables.

Variables	Chi-Square	Cramer's V
Companionship	61.30^{***}	0.40
Adverse Effects	41.50^{***}	0.33
Overreliance	34.29^{***}	0.30
Emotional Support	33.35^{***}	0.30
Non-judgemental	29.14^{***}	0.78
Assisting Self-reflection	28.25^{***}	0.27
Accessibility & Availability	17.47^{***}	0.21
Personalized Advice	15.41^{**}	0.20
Insufficient Depth and Emotional Connection	9.32^{*}	0.16
Technical & Privacy Issues	7.28	0.14
Lack of Human-like Interaction	3.82	0.10
Other Challenges	3.44	0.09
Lack of Professional Qualifications	2.76	0.08
Other Benefits	1.24	0.57

Note: Results are ordered in descending order by Chi-square value.

Table S5. Contingency Table between AI Applications and Assisting Self-reflection.

	Assisting Self-reflection (No)	Assisting Self-reflection (Yes)
Character-based Chatbot	96 (99.39)	16 (12.61)
LLMs	74 (85.19)	22 (10.81)
Others	124 (110.93)	1 (14.07)
Therapy Chatbots	45 (43.48)	4 (5.52)

Note: Values outside and inside of parentheses represent raw counts and expected counts, respectively.

Table S6. Contingency Table between AI Applications and Accessibility and Availability.

	Accessibility and Availability (No)	Accessibility and Availability (Yes)
Character-based Chatbot	91 (94.41)	21 (17.59)
LLMs	74 (80.92)	22 (15.08)
Others	119 (105.37)	6 (19.63)
Therapy Chatbots	38 (41.30)	11 (7.70)

Note: Values outside and inside of parentheses represent raw counts and expected counts, respectively.

Table S7. Contingency Table between AI Applications and Companionship.

	Companionship (No)	Companionship (Yes)
Character-based Chatbot	67 (92.94)	45 (19.06)
LLMs	86 (79.66)	10 (16.34)
Others	117 (103.73)	8 (21.27)
Therapy Chatbots	47 (40.66)	2 (8.34)

Note: Values outside and inside of parentheses represent raw counts and expected counts, respectively.

Table S8. Contingency Table between AI Applications and Emotional Support.

	Emotional Support (No)	Emotional Support (Yes)
Character-based Chatbot	68 (88.25)	44 (23.75)
LLMs	78 (75.64)	18 (20.36)
Others	112 (98.49)	13 (26.51)
Therapy Chatbots	43 (38.61)	6 (10.39)

Note: Values outside and inside of parentheses represent raw counts and expected counts, respectively.

Table S9. Contingency Table between AI Applications and Non-judgmental.

	Non-judgmental (No)	Non-judgmental (Yes)
Character-based Chatbot	92 (103.50)	20 (8.50)
LLMs	88 (88.71)	8 (7.29)
Others	124 (115.51)	1 (9.49)
Therapy Chatbots	49 (45.28)	0 (3.72)

Note: Values outside and inside of parentheses represent raw counts and expected counts, respectively.

Table S10. Contingency Table between AI Applications and Personalized Advice.

	Personalized Advice (No)	Personalized Advice (Yes)
Character-based Chatbot	101 (101.74)	11 (10.26)
LLMs	79 (87.20)	17 (8.80)
Others	122 (113.55)	3 (11.45)
Therapy Chatbots	45 (44.51)	4 (4.49)

Note: Values outside and inside of parentheses represent raw counts and expected counts, respectively.

Table S11. Contingency Table between AI Applications and Other Benefits.

	Other Benefits (No)	Other Benefits (Yes)
Character-based Chatbot	111 (111.41)	1 (0.59)
LLMs	96 (95.50)	0 (0.50)
Others	124 (124.35)	1 (0.65)
Therapy Chatbots	49 (48.74)	0 (0.26)

Note: Values outside and inside of parentheses represent raw counts and expected counts, respectively.

Table S12. Contingency Table between AI Applications and Adverse Effects.

	Adverse Effects (No)	Adverse Effects (Yes)
Character-based Chatbot	67 (92.94)	45 (19.06)
LLMs	86 (79.66)	10 (16.34)
Others	117 (103.73)	8 (21.27)
Therapy Chatbots	47 (40.66)	2 (8.34)

Note: Values outside and inside of parentheses represent raw counts and expected counts, respectively.

Table S13. Contingency Table between AI Applications and Insufficient Depth and Emotional Connection.

	Insufficient Depth and Emotional Connection (No)	Insufficient Depth and Emotional Connection (Yes)
Character-based Chatbot	100 (99.98)	12 (12.02)
LLMs	92 (85.70)	4 (10.30)
Others	110 (111.58)	15 (13.42)
Therapy Chatbots	39 (43.74)	10 (5.26)

Note: Values outside and inside of parentheses represent raw counts and expected counts, respectively.

Table S14. Contingency Table between AI Applications and Lack of Human-like Interaction.

	Lack of Human-like Interaction (No)	Lack of Human-like Interaction (Yes)
Character-based Chatbot	108 (106.72)	4 (5.28)
LLMs	92 (91.48)	4 (4.52)
Others	120 (119.11)	5 (5.89)
Therapy Chatbots	44 (46.69)	5 (2.31)

Note: Values outside and inside of parentheses represent raw counts and expected counts, respectively.

Table S15. Contingency Table between AI Applications and Lack of Professional Qualifications.

	Lack of Professional Qualifications (No)	Lack of Professional Qualifications (Yes)
Character-based Chatbot	109 (109.36)	3 (2.64)
LLMs	92 (93.74)	4 (2.26)
Others	124 (122.05)	1 (2.95)
Therapy Chatbots	48 (47.85)	1 (1.15)

Note: Values outside and inside of parentheses represent raw counts and expected counts, respectively.

Table S16. Contingency Table between AI Applications and Over-reliance.

	Over-reliance (No)	Over-reliance (Yes)
Character-based Chatbot	93 (105.26)	19 (6.74)
LLMs	93 (90.22)	3 (5.78)
Others	124 (117.47)	1 (7.53)
Therapy Chatbots	49 (46.05)	0 (2.95)

Note: Values outside and inside of parentheses represent raw counts and expected counts, respectively.

Table S17. Contingency Table between AI Applications and Technical & Privacy Issues.

	Technical & Privacy Issues (No)	Technical & Privacy Issues (Yes)
Character-based Chatbot	104 (107.90)	8 (4.10)
LLMs	92 (92.48)	4 (3.52)
Others	123 (120.42)	2 (4.58)
Therapy Chatbots	49 (47.20)	0 (1.80)

Note: Values outside and inside of parentheses represent raw counts and expected counts, respectively.

Table S18. Contingency Table between AI Applications and Other Challenges.

	Other Challenges (No)	Other Challenges (Yes)
Character-based Chatbot	112 (111.41)	0 (0.59)
LLMs	96 (95.50)	0 (0.50)
Others	124 (124.35)	1 (0.65)
Therapy Chatbots	48 (48.74)	1 (0.26)

Note: Values outside and inside of parentheses represent raw counts and expected counts, respectively.

Table S19. Contingency Table between AI Applications and Community Type.

	AI Community	Mental Health Community
Character-based Chatbot	80 (57.47)	32 (54.53)
LLMs	81 (49.26)	15 (46.74)
Others	19 (64.14)	106 (60.86)
Therapy Chatbots	16 (25.14)	33 (23.86)

Note: Values outside and inside of parentheses represent raw counts and expected counts, respectively.

Figure S1. Distribution of Benefits between Posts from Users With and Without AI Mental Health Experiences.

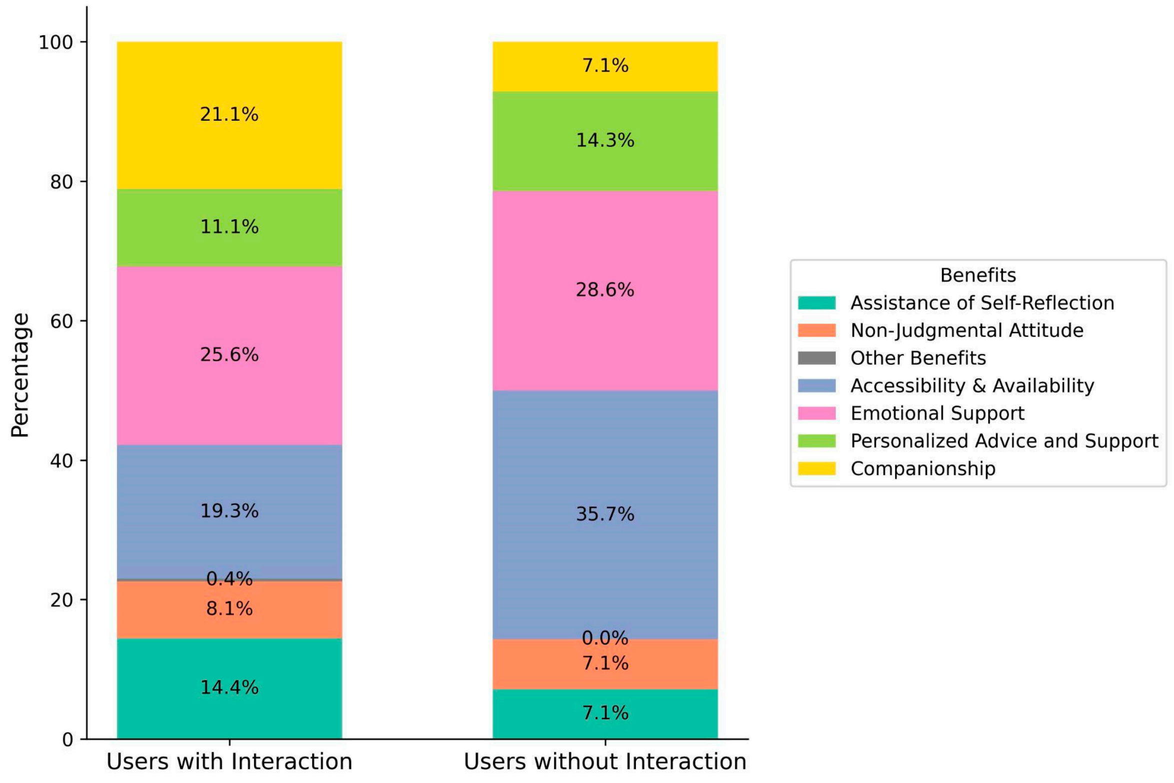

Figure S2. Distribution of Challenges between Posts from Users With and Without AI Mental Health Experiences.

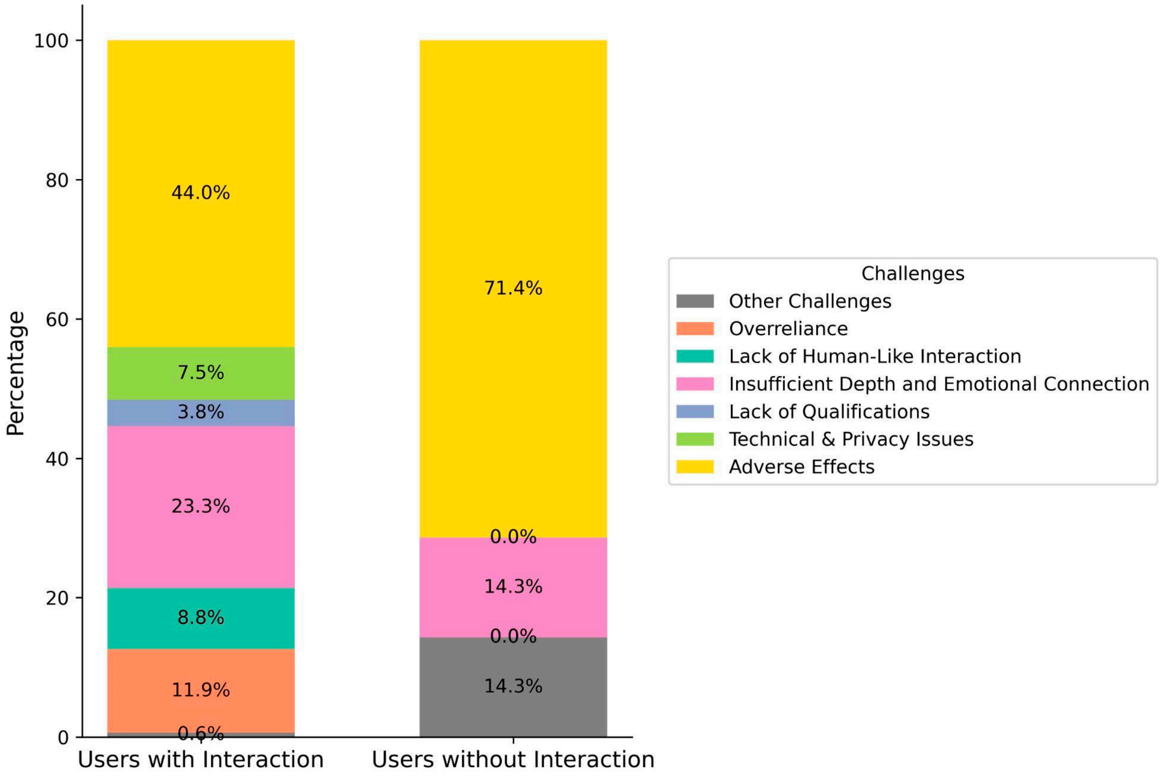

Figure S3. Distribution of Benefits between Posts from AI and Mental Health Communities.

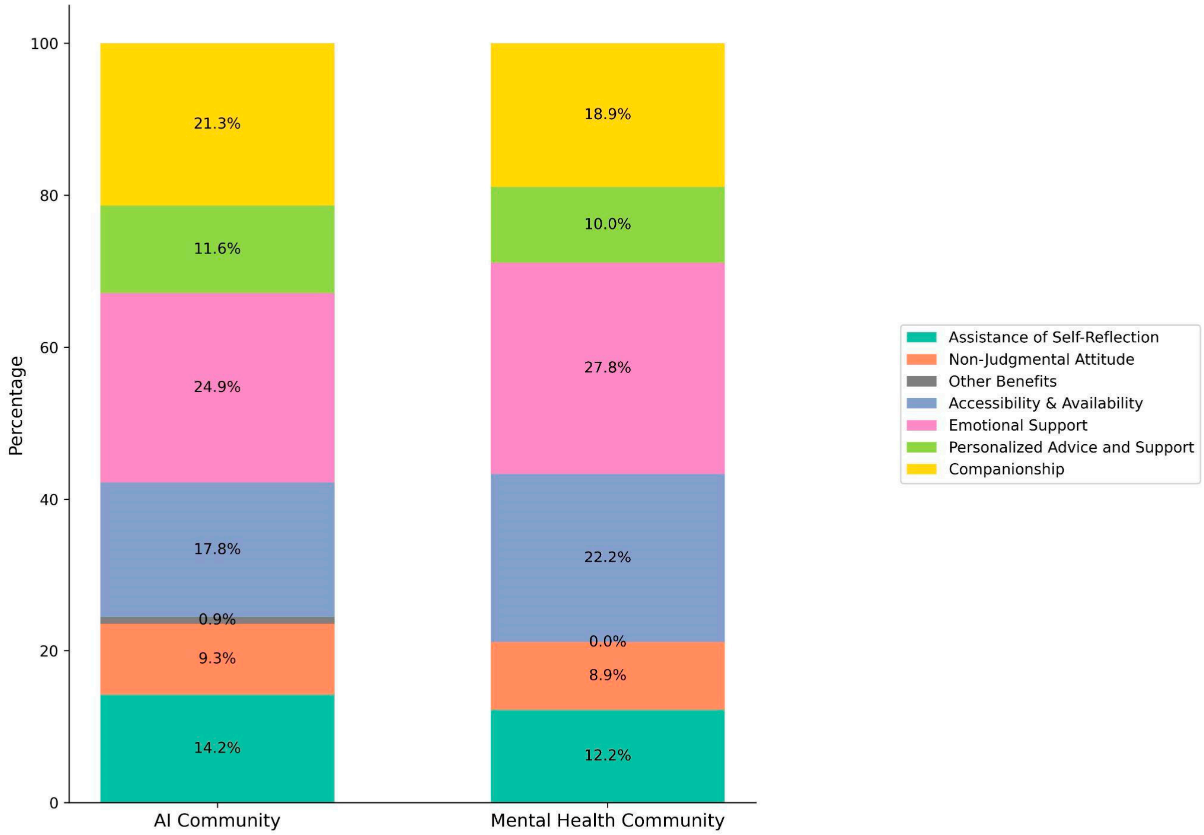

Figure S4. Distribution of Challenges between Posts from AI and Mental Health Communities.

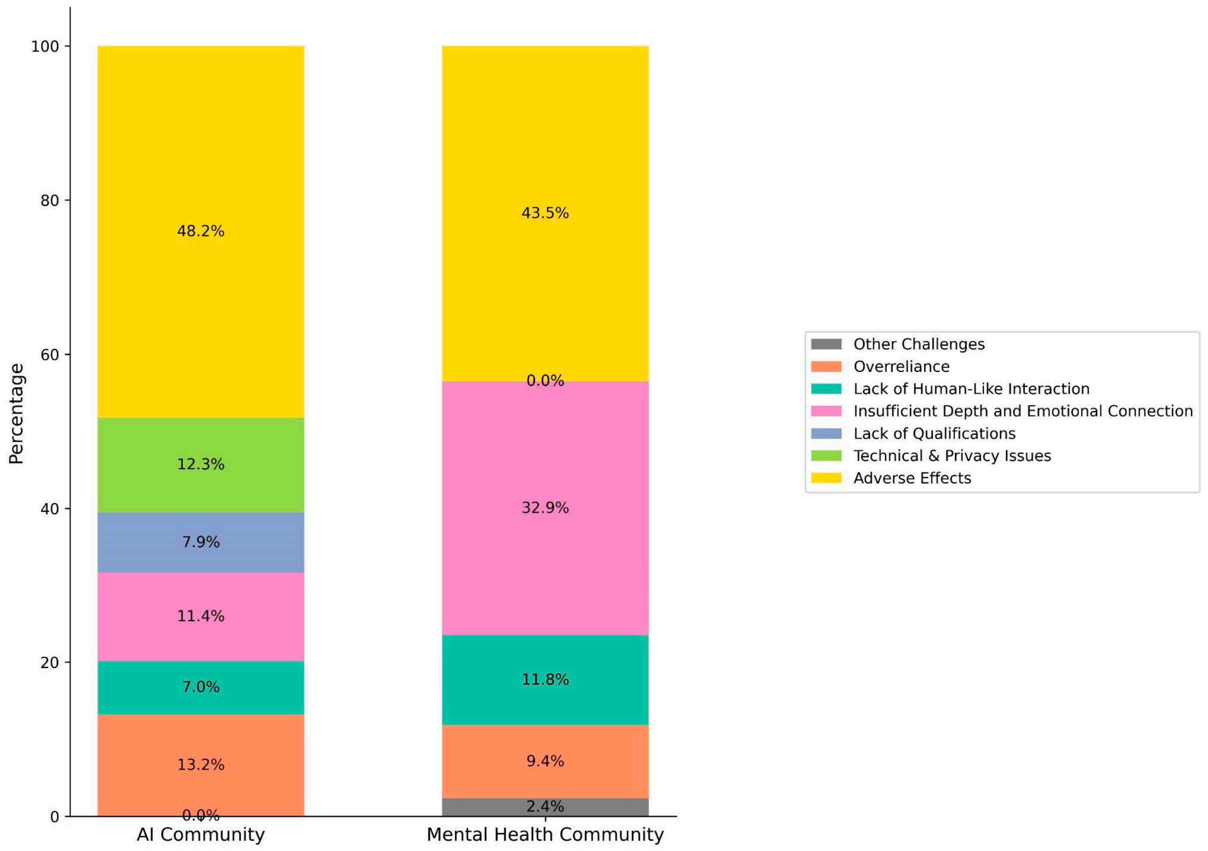

Supplement: Supplementary file 1 [file behavsci-15-01172-s001.zip › behavsci-3747895-supplementary.pdf]
